# Antimicrobial resistance in hypermucoviscous and non-hypermucoviscous *Klebsiella pneumoniae*: a systematic review and meta-analysis

**DOI:** 10.1080/22221751.2024.2438657

**Published:** 2024-12-04

**Authors:** Hiroki Namikawa, Ken-Ichi Oinuma, Yukihiro Kaneko, Hiroshi Kakeya, Taichi Shuto

**Affiliations:** a Department of Medical Education and General Practice, Graduate School of Medicine, Osaka Metropolitan University, Osaka, Japan; b Department of Bacteriology, Graduate School of Medicine, Osaka Metropolitan University, Osaka, Japan; c Research Center for Infectious Disease Sciences, Graduate School of Medicine, Osaka Metropolitan University, Osaka, Japan; d Department of Infection Control Science, Graduate School of Medicine, Osaka Metropolitan University, Osaka, Japan

**Keywords:** *Klebsiella pneumoniae*, hypermucoviscous, antimicrobial resistance, extended-spectrum beta-lactamase, carbapenem-resistant

## Abstract

Antimicrobial resistance has recently increased due to emerging carbapenem-resistant *Klebsiella pneumoniae* and extended-spectrum β-lactamase (ESBL)-producing strains of *K. pneumoniae*, especially among hypermucoviscous *K. pneumoniae* (hmKp) strains. To evaluate the prevalence of ESBL-producing and carbapenem-resistant strains in hmKp and non-hmKp clinical isolates through a systematic review and meta-analysis. We searched PubMed, Scopus, and Cochrane Library databases from January 2000 to June 2023. Clinical and in vivo/in vitro studies involving confirmed *K. pneumoniae* clinical isolates differentiated into hmKP and non-hmKP strains based on string test results. Odds ratios (ORs) and 95% confidence intervals (CIs) were calculated based on the number of individuals in each target group. Forest plots were used to visualize the effect sizes and 95% CIs of individual studies estimated using the inverse variance and DerSimonian – Laird methods with fixed – and random-effects models, respectively. Heterogeneity was assessed using Cochran’s Q test (*I^2^* ≥ 50%). Fifteen studies comprising 2049 clinical isolates of *K. pneumoniae* met the inclusion criteria. Meta-analysis revealed that hmKp strains were associated with a significantly lower prevalence of ESBL-producing strains (pooled OR: 0.26, 95% CI: 0.11–0.63, *P* = 0.003) and a slightly lower prevalence of carbapenem-resistant strains than non-hmKp strains (pooled OR: 0.63, 95% CI: 0.40–0.97, *P* = 0.038). hmKp strains exhibited lower and slightly lower prevalence of ESBL production and carbapenem resistance, respectively, than non-hmKp strains. However, given the rising prevalence of ESBL-producing and carbapenem-resistant hmKp strains, patients infected by string-test-positive *K. pneumoniae* must be managed prudently, considering the potential for highly resistant strains.

## Introduction

*Klebsiella pneumoniae*, a Gram-negative bacterium, is well known for its adaptability as a commensal organism within the human microbiota and its capacity to provoke a wide array of infections, including urinary tract, intra-abdominal, and bloodstream infections and pneumonia. The emergence of carbapenem-resistant *K. pneumoniae* (CRKP) and extended-spectrum β-lactamase (ESBL)-producing strains of *K. pneumoniae* has become a significant public health concern worldwide [1]. Recently, there has been notable interest in the unique hypermucoviscous (string-test-positive) phenotype of *K. pneumoniae* due to its link with severe and invasive infections, which frequently result in metastatic dissemination and unfavourable clinical outcomes [2]. Hypermucoviscous *K. pneumoniae* (hmKp) is defined by its ability to generate a dense, viscous capsule that grants it enhanced virulence attributes, including resistance to phagocytosis and the formation of biofilms [3]. HmKp has been implicated in various infections, spanning from community-acquired pyogenic liver abscesses to healthcare-associated bloodstream infections, posing a significant clinical challenge.

hmKp was previously believed to have a lower resistance rate to nearly all clinically used antimicrobial agents than non-hmKp strains. However, recent years have seen increasing antimicrobial resistance among hmKp strains, posing challenges to traditional therapeutic strategies. In particular, ESBL-producing and carbapenem-resistant strains are increasingly prevalent within the hmKp population [4–7]. ESBLs are enzymes capable of hydrolysing a wide range of beta-lactam antibiotics, such as oxyimino-cephalosporins and monobactams, rendering them ineffective. Carbapenem resistance results from various mechanisms, including the action of carbapenemases (enzymes that hydrolyze carbapenem antibiotics), decreased permeability of bacterial cell membranes, upregulation of efflux pumps, and alterations in the bacterial cell wall structure. These mechanisms collectively diminish the effectiveness of carbapenem antibiotics [8]. ESBL-producing and carbapenem-resistant strains pose a significant challenge in clinical settings by limiting treatment options, increasing the risk of treatment failure, and facilitating the spread of resistant strains.

Understanding the disparities in antimicrobial resistance between hmKp and non-hmKp strains is pivotal in effectively managing *K. pneumoniae* infections and curbing the dissemination of resistant strains. Although we conducted a thorough literature search, we did not identify any meta-analyses on this subject. Through this systematic review and meta-analysis, we assessed the prevalence of ESBL-producing and carbapenem-resistant strains in hmKp and non-hmKp clinical isolates.

## Methods

### Literature review

This systematic review and meta-analysis adhered to the Preferred Reporting Items for Systematic Reviews and Meta-Analyses (PRISMA) guidelines. A research model was developed based on a framework established previously [9], with minor modifications. A comprehensive literature search was conducted using the PubMed, Scopus, and Cochrane Library databases to retrieve publications from January 2000 to June 2023. The search terms used were (1) “*Klebsiella pneumoniae*” or “*K. pneumoniae*”, (2) “hypermucoviscous’ or “hypermucoviscosity”, and (3) “resistant” or “resistance”. Based on the inclusion criteria, full-text articles in English encompassing clinical trials, cohort, case–control, and cross-sectional studies involving confirmed *K. pneumoniae* clinical isolates, as well as in vivo/in vitro studies utilizing such isolates, were considered. Only studies employing a string test to differentiate between hmKP and non-hmKP strains were eligible for inclusion in the present systematic review. Reviews, systematic reviews, meta-analyses, guidelines, editorials, letters to the editor, comments, case reports, pediatric studies, studies with fewer than 10 patients per group, and studies focusing solely on drug-resistant strains were excluded. The primary outcome measure was the prevalence of ESBL-producing and carbapenem-resistant strains confirmed through antimicrobial susceptibility testing. Carbapenem-resistant strains were defined based on resistance to imipenem or meropenem. When resistance rates for both carbapenem drugs were available, data indicating a higher resistance rate were selected for analysis.

### Data extraction and quality assessment

Two reviewers independently extracted data from the literature using a standardized format. The following data were extracted as key variables: country, study design, publication year, duration, number of patients with/clinical isolates of *K. pneumoniae* (hmKp and non-hmKp), and prevalence of ESBL-producing and carbapenem-resistant strains. Any inconsistencies or disagreements between reviewers were resolved through discussion and consensus. Quality assessment was conducted using the Newcastle-Ottawa Scale (NOS).

### Statistical analysis

A meta-analysis was conducted to summarize the differences in the prevalence of ESBL-producing and carbapenem-resistant strains in hmKp and non-hmKp isolates based on the data extracted from the studies included in the systematic review. Odds ratios (ORs) and 95% confidence intervals (CIs) were calculated based on the number of individuals in each target group. Forest plots were used to visualize the effect sizes and 95% CIs of individual studies, which were estimated using the inverse variance and DerSimonian–Laird methods with fixed- and random-effects models, respectively. Heterogeneity was assessed using Cochran’s Q test, with *I*^2^ ≥ 50% indicating statistical heterogeneity. Publication bias and small-study effects were examined by visually assessing funnel plots and Egger’s test. A two-tailed *P* <0.05 was regarded as statistically significant. When high heterogeneity was article, the causal literature was identified on *I*^2^ value basis. A sensitivity analysis was then performed on a group of articles that excluded studies from the same background as the article with high heterogeneity. The meta-analysis model for the sensitivity analysis was performed with the same settings as the overall analysis model. All statistical analyses were performed using EZR, a modified version of the R commander that includes statistical functions commonly used in biostatistics.

## Results

### Study selection and characteristics

**Figure 1.**
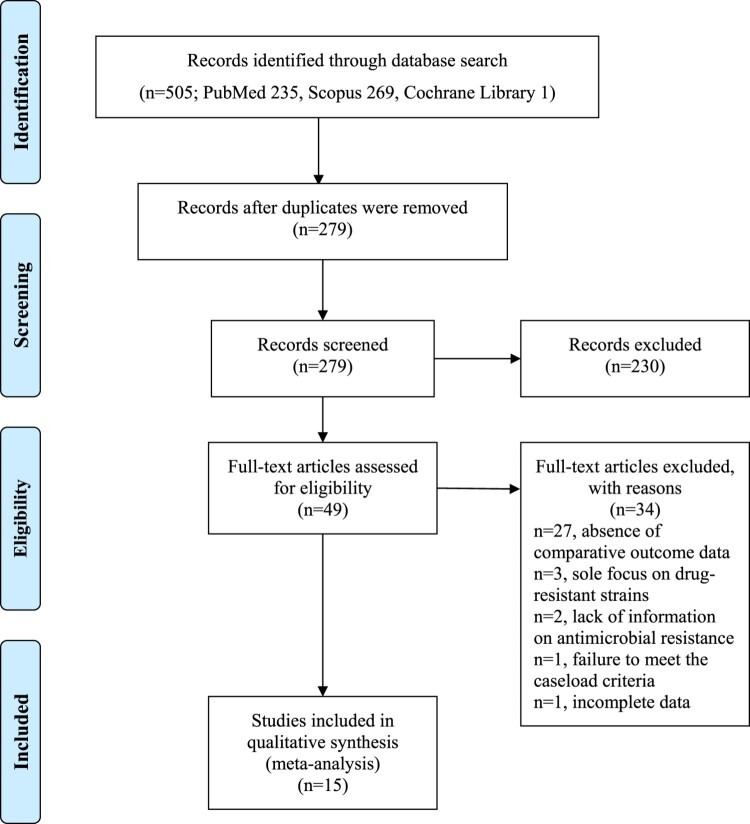
Flow diagram illustrating the process of selecting the studies included in this review.

Figure 1 depicts a PRISMA flow diagram illustrating the study selection process. The initial search yielded 505 relevant published studies (PubMed, *n* = 235; Scopus, *n* = 269; Cochrane Library, *n* = 1). After removing 226 duplicates, the titles and abstracts of the remaining 279 articles were screened. Among them, 230 articles were excluded because they did not meet the study design or outcome criteria. Subsequently, 49 papers underwent a full-text review, and 34 were excluded for the following reasons: absence of comparative outcome data (*n* = 27), sole focus on drug-resistant strains (*n* = 3), lack of information on antimicrobial resistance (*n* = 2), failure to meet the caseload criteria (*n* = 1), and incomplete data (*n* = 1) (Figure 1). Following a thorough examination of the full texts, 15 studies met the inclusion criteria for this review [10–24]. The key characteristics of the selected studies are summarized in Table 1. These articles were published between 2014 and 2023 and had been conducted in seven countries spanning four continents: China, Egypt, Iran, Japan, Pakistan, Spain, and the USA, representing Asia, Africa, Europe, and North America. All studies were observational, with seven being retrospective, while the nature of the remaining eight studies was unclear. Sample sizes ranged from 37 to 428, totalling 2049 clinical isolates of *K. pneumoniae*, of which 568 (27.7%) were hmKp clinical isolates. Nine studies analyzed the prevalence of ESBL-producing strains, whereas 14 analyzed the prevalence of carbapenem-resistant strains. The prevalence of ESBL-producing strains among the hmKp and non-hmKp strains ranged from 0% to 70.6% and from 8.9% to 70.6%, respectively. Similarly, the prevalence of carbapenem-resistant strains in the hmKp and non-hmKp groups ranged from 0% to 55.6% and from 1.1% to 70.2%, respectively. In one study [21], the data on meropenem resistance appeared inadequate, leading us to use data on imipenem resistance instead. The NOS scores are summarized in Table 2. All the studies included in this review scored between 5 and 6, indicating moderate quality. The main areas of weakness were the selection of controls and comparability between groups.

**Table 1.** Characteristics of the studies included in the systematic review and meta-analysis.

Author (year)	Country	Design	Period	Population or clinical isolates (hmKp: N)	ESBL		Carbapenem-resistant	
					hmKp	non-hmKp	hmKp	non-hmKp
Li (2014) [10]	China	Retrospective observational	2010–2012	88 patients with Kp infection (hmKp: 29)	5 (17.2%)	33 (55.9%)	0 (0%)	5 (8.5%)
Guo (2016) [11]	China	Retrospective observational	2012–2014	43 patients with Kp VAP (hmKp: 14)	2 (14.3%)	18 (62.1%)	0 (0%)	1 (3.4%)
Gharrah (2017) [12]	Egypt	Observational	No data	100 Kp clinical isolates (hmKp: 32)	2 (6.3%)	48 (70.6%)	no data	No data
Eghbalpoor (2019) [13]	Iran	Observational	2017	140 patients with Kp UTI (hmKp: 17)	12 (70.6%)	36 (29.3%)	5 (29.4%)	12(9.8%)
Namikawa (2019) [14]	Japan	Retrospective case-control	2012–2018	114 patients with Kp bacteremia (hmKp: 24)	0 (0%)	8 (8.9%)	0 (0%)	1 (1.1%)
Rastegar (2019) [15]	Iran	Observational	2017–2018	146 Kp clinical isolates (hmKp: 22)	No data	No data	2 (9.1%)	47 (37.9%)
Ding (2020) [16]	China	Retrospective observational	2017–2018	37 Kp clinical isolates with RI (hmKp: 16)	No data	No data	0 (0%)	2 (9.5%)
Lin (2020) [17]	China	Observational	2010–2016	428 Kp clinical isolates (hmKp: 109)	20 (18.3%)	161 (50.5%)	9 (8.3%)	33 (10.3%)
Imtiaz (2021) [18]	Pakistan	Observational	No data	200 Kp clinical isolates (hmKp: 43)	No data	No data	15 (34.9%)	60 (38.2%)
Elbrolosy (2021) [19]	Egypt	Observational	2019–2020	84 clinical isolates with Kp infections (hmkp: 27)	5 (18.5%)	34 (59.6%)	15 (55.6%)	40 (70.2%)
Ballén (2021) [20]	Spain	Observational	2016–2017	127 Kp clinical isolates (hmKp: 17)	6 (35.3%)	49 (44.5%)	2 (11.8%)	12 (10.9%)
Yang (2022) [21]	China	Retrospective cohort	2017–2021	139 patients with Kp infection (hmKp: 86)	no data	no data	32 (37.2%)	29 (54.7%)
Kochan (2022) [22]	USA	Retrospective observational	2015–2017	104 patients with Kp bacteremia (hmKp: 10)	no data	no data	0 (0%)	9 (9.6%)
Jin (2023) [23]	China	Retrospective observational	2018–2021	203 patients with Kp infection (hmKp: 90)	no data	no data	1 (1.1%)	13 (11.5%)
Dan (2023) [24]	China	Observational	2018–2019	96 Kp clinical isolates (hmKp: 32)	5 (15.6%)	28 (43.8%)	4 (12.5%)	13 (20.3%)

ESBL: extended spectrum beta-lactamase, hmKp: hypermucoviscous *Klebsiella pneumoniae*, *N*: sample size, RI: respiratory infection, UTI: urinary tract infection, VAP: ventilator-associated pneumonia.

**Table 2.** Quality assessment using the using the Newcastle–Ottawa Scale.

Author (year)	Selection				Comparability	Exposure			Total
	Q1	Q2	Q3	Q4	Q5	Q6	Q7	Q8	
Li (2014) [10]	⋆			⋆		⋆	⋆	⋆	5
Guo (2016) [11]	⋆			⋆		⋆	⋆	⋆	5
Gharrah (2017) [12]	⋆			⋆		⋆	⋆	⋆	5
Eghbalpoor (2019) [13]	⋆	⋆		⋆		⋆	⋆	⋆	6
Namikawa (2019) [14]	⋆			⋆		⋆	⋆	⋆	5
Rastegar (2019) [15]	⋆	⋆		⋆		⋆	⋆	⋆	6
Ding (2020) [16]	⋆			⋆		⋆	⋆	⋆	5
Lin (2020) [17]	⋆			⋆		⋆	⋆	⋆	5
Imtiaz (2021) [18]	⋆	⋆		⋆		⋆	⋆	⋆	6
Elbrolosy (2021) [19]	⋆	⋆		⋆		⋆	⋆	⋆	6
Ballén (2021) [20]	⋆	⋆		⋆		⋆	⋆	⋆	6
Yang (2022) [21]	⋆					⋆	⋆	⋆	5
Kochan (2022) [22]	⋆			⋆		⋆	⋆	⋆	5
Jin (2023) [23]	⋆			⋆		⋆	⋆	⋆	5
Dan (2023) [24]	⋆			⋆		⋆	⋆	⋆	5

### Meta-analysis

**Figure 2.**
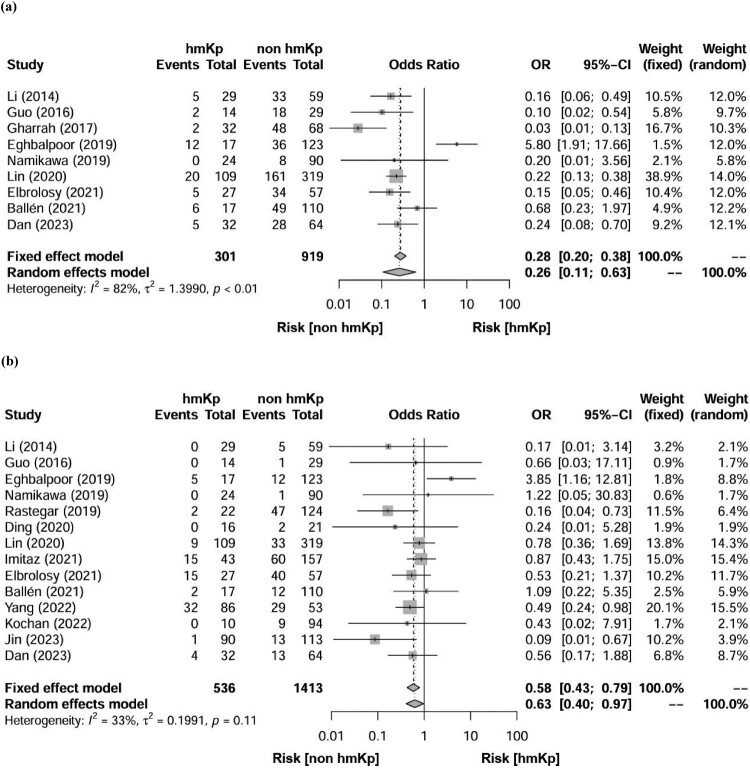
Forest plot of the odds ratios for differences in the prevalence of (a) extended-spectrum β-lactamase-producing and (b) carbapenem-resistant strains in clinical isolates of hypermucoviscous *Klebsiella pneumoniae* (hmKp) and non-hmKp.

**Figure 3.**
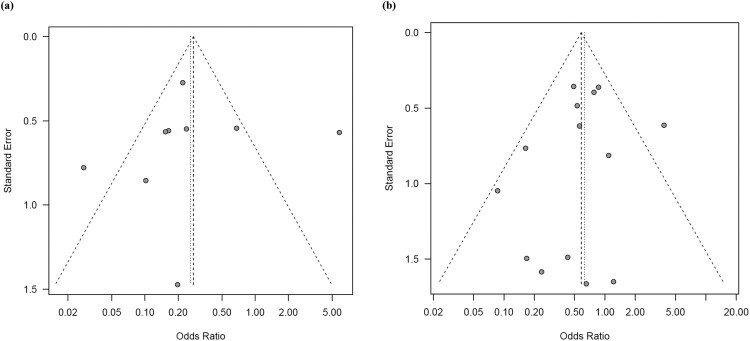
Funnel plot of the odds ratios for (a) extended-spectrum β-lactamase-producing and (b) carbapenem-resistant strains.

Regarding the analysis of ESBL-producing strains, data were available from 9 studies encompassing 1220 clinical isolates, of which 301 and 919 were hmKp and non-hmKp strains, respectively. Among these, 57 (18.9%) hmKp strains and 415 (45.2%) non-hmKp strains produce ESBL. The funnel plot showed visual symmetry (Figure 3a) and the Egger's test was also statistically non-significant (*P* = 0.8859), suggesting no publication bias. Meta-analysis revealed that hmKp strains were associated with a significantly lower prevalence of ESBL-producing strains than non-hmKp strains (pooled OR: 0.26, 95% CI: 0.11–0.63, *P* = 0.003) (Figure 2a). The *I*^2^ was 82%, indicating high heterogeneity among the studies (Q = 44.34, *P* < 0.0001). The search for the literature responsible for the increased heterogeneity led to the identification of “Eghbalpoor 2019” (*I*^2^ value decreased to 45% after exclusion of this literature). Two characteristics of this study were considered: (1) national and regional characteristics (Middle East region) and (2) small sample size of highly viscous pneumococci (*n* = < 20). Based on the above, two sensitivity analyses were performed in this study: one was a meta-analysis that excluded literature from the Middle East region. The second was a meta-analysis that excluded literature with a sample size of less than 20. The meta-analysis excluding the Middle East region (6 articles included) resulted in a pooled OR of 0.24 [95%CI: 0.16–0.35] and *I*^2^ was 5%. The meta-analysis that excluded references with small sample sizes (6 articles included) resulted in a pooled OR of 0.17 [95%CI: 0.11–0.27] and *I*^2^ was 25%. These results approximated the meta-analysis results for all articles, suggesting the robustness of the present results.

Regarding the analysis of carbapenem-resistant strains, data were available from 14 studies comprising 1949 clinical isolates, with 536 hmKp strains and 1413 non-hmKp strains. Among these, 85 (15.9%) hmKp strains and 277 (19.6%) non-hmKp strains were carbapenem-resistant. The funnel plot showed visual symmetry (Figure 3b) and the Egger's test was also statistically non-significant (*P* = 0.1883), suggesting no publication bias. Meta-analysis revealed that hmKp strains were associated with a slightly lower prevalence of carbapenem-resistant strains than non-hmKp strains (pooled OR: 0.63, 95% CI: 0.40–0.97, *P* = 0.038) (Figure 2b). The *I*^2^ was 33% (Q = 19.46, *P* = 0.11), indicating no statistically significant heterogeneity among the studies.

## Discussion

This systematic review identified 15 observational studies published between 2014 and 2023, comprising 2049 clinical isolates of *K. pneumoniae*, which is approximately five times the sample size of the largest study included in the meta-analysis. The meta-analysis revealed that hmKp strains displayed a significantly lower prevalence of ESBL-producing strains and a slightly lower prevalence of carbapenem-resistant strains than non-hmKp strains.

Consistent with the articles included in this review, a few studies have reported a significantly lower prevalence of ESBL-producing strains among hmKp strains than among non-hmKp strains [25,26]. Some strains of *K. pneumonia*e possess the clustered regularly interspaced short palindromic repeats (CRISPR) and CRISPR-associated (Cas) system, which limits the horizontal transmission of ESBL or carbapenemase genes by preventing the acquisition of foreign DNA from plasmids and bacteriophages [27,28]. Although the precise cause of the low incidence of ESBL-producing hmKp strains remains uncertain, it is possible that hmKp possesses this inherent CRISPR-Cas system to evade antibiotic resistance. However, the number of reports on ESBL-producing hmKp strains appears to be increasing globally every year [3,29,30]. Furthermore, Tanimoto et al. demonstrated a significantly different distribution of specific ESBL genes and plasmid types between hmKp and non-hmKp strains, suggesting that the transmission routes of ESBL genes may vary between the two groups of strains [5]. Further exploration of the genetic and molecular mechanisms driving this phenomenon may yield valuable insights into the correlation between mucoviscosity and ESBL production. Currently, the frequency of ESBL-producing strains between hmKp and non-hmKp strains is significantly different. However, as the number of ESBL-producing strains is likely to increase, careful management of hmKp infections is required.

Consistent with the articles included in this review, Zhou et al. reported a lower prevalence of carbapenem-resistant hmKp strains than among non-hmKp strains [31]. Until about a decade ago, hmKp was generally considered susceptible to common antimicrobials, including carbapenems [30]. However, the recent widespread dissemination of mobile genetic elements encoding carbapenemases has resulted in the increasing incidence of carbapenem-resistant hmKp isolates [7,32]. Recent studies have reported the prevalence of carbapenem-resistant hmKp strains as 7.5%–19.6% of all CRKP strains [7,33–35]. In this study, the prevalence of carbapenem-resistant strains was only slightly lower in hmKp strains than in non-hmKp strains, and the disparity was not as pronounced as that observed in ESBL-producing strains. Wang et al. found that carbapenem-resistant hmKp was significantly associated with various risk factors, including prior antibiotic therapy, previous hospitalization, and invasive procedures [7]. Infections by CRKP, including hmKp strains, are currently linked to high mortality rates, thus representing major threats to global public health [6]. Consistent with this prevailing opinion, Xu et al. estimated the mortality rate of patients infected with CRKP at approximately 42% through their systematic review and meta-analysis [36]. Furthermore, therapeutic options for CRKP infections are extremely limited due to the scarcity of effective antibacterial drugs. Therefore, it is crucial to consider the risks posed by CRKP strains when administering treatment to patients with hmKp infections and a history of the aforementioned risk factors.

This study has several limitations. First, we limited our search to three databases, namely, PubMed, Scopus, and the Cochrane Library, which resulted in a smaller number of records. It is possible that relevant studies from other sources were not included. Secondly, our inclusion criteria were restricted to articles published in English, which may have limited the scope of our analysis. Third, our review included only observational studies, many of which had a retrospective design, potentially introducing selection bias and affecting the reliability of the results. Fourth, the geographical distribution of the studies was predominantly limited to certain regions, particularly Asia (11/15 studies), and data from other parts of the world were lacking. This may have affected the generalizability of the findings. Fifth, we focused only on clinical strains of *K. pneumoniae* isolated from humans in this study. Future research should also investigate *K. pneumoniae* strains originating from animals and the environment. Finally, although this study provides insights into the prevalence of ESBL-producing and carbapenem-resistant strains among hmKp and non-hmKp isolates, it does not delve into the genetic and molecular mechanisms underlying the differences in resistance patterns. Further research involving well-designed prospective studies collecting data from more countries on different continents and molecular analyses is necessary to fully understand the factors that contribute to antimicrobial resistance in hmKp and non-hmKp strains.

In conclusion, the present study demonstrated that hmKp strains exhibited a lower prevalence of ESBL production and a slightly lower prevalence of carbapenem resistance than non-hmKp strains. However, considering the increasing prevalence of ESBL-producing hmKp strains, which may continue to rise, and the almost equivalent prevalence of carbapenem-resistant hmKp and non-hmKp strains, patients infected by string-test-positive *K. pneumoniae* must be managed prudently, taking into account the potential for highly resistant strains. Further research is essential to elucidate the genetic and molecular mechanisms underlying hmKp antimicrobial resistance and to continuously monitor the evolution of these resistance patterns.

## Data Availability

All data generated or analyzed during this study are included in this published article.
